# Cost-Sensitive Uncertainty Hypergraph Learning for Identification of Lymph Node Involvement With CT Imaging

**DOI:** 10.3389/fmed.2022.840319

**Published:** 2022-02-10

**Authors:** Qianli Ma, Jielong Yan, Jun Zhang, Qiduo Yu, Yue Zhao, Chaoyang Liang, Donglin Di

**Affiliations:** ^1^Department of Thoracic Surgery, China-Japan Friendship Hospital, Beijing, China; ^2^The School of Software, Tsinghua University, Beijing, China; ^3^Tencent AI Lab, Shenzhen, China

**Keywords:** lymph node involvement, CT imaging, hypergraph learning, cost-sensitive, lung cancer

## Abstract

Lung adenocarcinoma (LUAD) is the most common type of lung cancer. Accurate identification of lymph node (LN) involvement in patients with LUAD is crucial for prognosis and making decisions of the treatment strategy. CT imaging has been used as a tool to identify lymph node involvement. To tackle the shortage of high-quality data and improve the sensitivity of diagnosis, we propose a Cost-Sensitive Uncertainty Hypergraph Learning (CSUHL) model to identify the lymph node based on the CT images. We design a step named “Multi-Uncertainty Measurement” to measure the epistemic and the aleatoric uncertainty, respectively. Given the two types of attentional uncertainty weights, we further propose a cost-sensitive hypergraph learning to boost the sensitivity of diagnosing, targeting task-driven optimization of the clinical scenarios. Extensive qualitative and quantitative experiments on the real clinical dataset demonstrate our method is capable of accurately identifying the lymph node and outperforming state-of-the-art methods across the board.

## 1. Introduction

**Figure 1:**
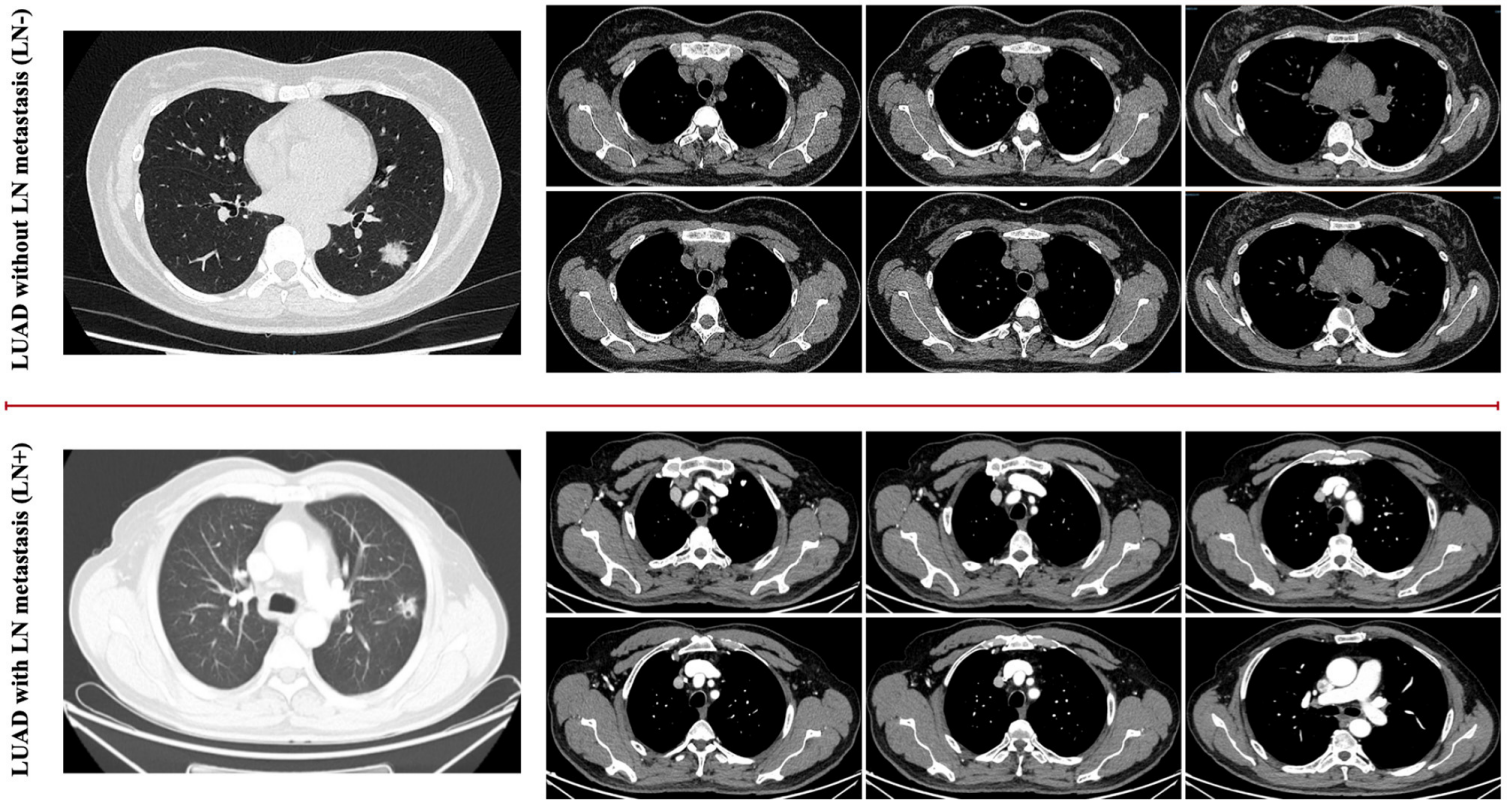
It is difficult for humans to identify the difference between the LUAD with LN metastasis cases as well as the LUAD without metastasis cases based on the general visualized CT images, as shown in the examples for comparison.

Lung cancer is the most commonly diagnosed cancer and the leading cause of cancer death worldwide (1, 2). About 2.1 million new lung cancer cases and 1.8 million deaths were predicted in 2018 (3). In 2020, these two numbers rise to 2.2 million and 1.8 million, respectively, (2). Lung adenocarcinoma (LUAD) is the most common type of lung cancer (4–6). The presence of metastasis in the lymph nodes (7) is an important prognostic factor in lung cancer. Accurate identification of lymph node (LN) involvement in patients with LUAD, as shown in Figure 1, is crucial for prognosis and treatment strategy decisions (8, 9). Patients without metastatic lymph nodes, or with only intrapulmonary or hilar lymph nodes, are generally considered candidates for straightforward resection. Although the sub-types of LUAD are found related to the predictors of LN metastasis, they are available postoperatively (10). Information of the preoperative LN metastasis is valuable for the adequacy of surgical resection and the decision of the adjuvant therapy (11). The accurate prediction of pathologic stage for patients with lung cancer is of utmost importance. Pathologic tumor stage is considered a pivotal factor relating to survival in NSCLC, and the 5-year survival rates vary from 83% in pathological stage IA to 23% in stage IIIA tumors (12). Computed tomography (CT) is commonly used for the evaluation of pulmonary nodules (13, 14). Many studies were designed to determine whether pulmonary nodules are benign or malignant. Zhong et al. (15) propose to use relief-based feature method and support vector machine to evaluate the impact of radiomics features in predicting the prognosis of occult mediastinal lymph node metastasis in lung adenocarcinoma. Dai et al. (16) find that lymph node micrometastases are more frequently seen in adenocarcinomas with a micropapillary component, which could give suggestive prognostic information to patients with stage I resected lung adenocarcinoma with a micropapillary component. CT has been widely used as a noninvasive diagnostic modality for diagnosis, clinical staging, survival prediction, surveillance of therapeutic response in lung cancer patients (17, 18). However, few studies have used chest CT to explore whether lymph node metastasis in LUAD (19). Therefore, in order to make a better decision on the prognosis and treatment strategies of lung cancer, as well as more fully grasp the information of lymph nodes, in this work, we utilize CT to predict whether LUAD has lymph node metastasis.

There are two main challenges of identifying the lymph node with CT imaging, listed below, that motivate our approach.

1. Noisy data, due to the collection of clinical CT images using different reconstruction kernels and CT manufacturers, along with possible patient movements;
2. The reliable sensitivity of diagnosis is relatively more important and meaningful than other criteria in the clinical scenario.

For the first challenge, a few current research works are proposed to tackle the issue of clinical data quality, mainly focusing on noise and artifact reduction, super resolution and other aspects (20). Zhang and Yu (21) propose to train their convolutional neural network using virtual metal-inserted CT images, targeting on the noise of metal artifacts. Tan et al. (22) further utilizes the SRGAN neural network to reconstruct super resolution images from the original chest CT images to improve the resolution and ultimately improve the classification results of COVID-19. Due to the scale of available data in this task being limited, we adopt the two uncertainty measurements (23) to improve the quality of pathological representations, *i.e*., epistemic and the aleatoric uncertainty, respectively, generated by the “Classifier Measuring” and “Statistical Measuring.” In this manner, our model is capable of allocating the different attentional weights combined with the two uncertainty measurements. Due to ignoring the underlying correlation between samples, some machine learning methods such as Random forest, Boosting, or CNN are lacking in effect, but graph learning and further hypergraph learning methods can make up for this deficiency. Hypergraph Learning methods (24–26) perform well on generate the high-order representations for complex data, such as whole-slide images (WSI) (27), CT imaging (23), drug-target interactions (28), *etc*. Therefore, given the data with uncertainty weights, we further propose an uncertainty hypergraph learning to extract the high-order representations from the CT images, which augments the pathological informative features effectively.

With regards to the second challenge, several works have made efforts on lymph node involvement. Zhou et al. (29) studied one or a combination of machine learning methods in Logistic regression, Random forest, XGBoost, and GBDT to construct lymph node metastasis in patients with poorly differentiated intramucosal gastric cancer. Supervised machine learning methods including random forest classifier, artificial neural network, decision tree, gradient boosting decision tree, extreme gradient boosting, and adaptive boosting can also be used to predict central lymph node metastasis in patients with papillary thyroid cancer (30). Besides improving the accuracy of overall prediction, we further focus on boosting the performance on sensitivity by the designed “Two-Stage Cost Sensitive Hypergraph Learning.” One stage is to capture the cost sensitivity of negative cases in the latent feature spaces, which will enable the hypergraph model to allocate the lymph node involvement cases more weights. The other is called “Supervising Cost Sensitivity,” making the loss function supervise the hypergraph model with more attached importance on the patients with LUAD for individual preoperative prediction of LN metastasis. Combining the structures introduced above, the overall framework we proposed, named as “Cost-Sensitive Uncertainty Hypergraph Learning (CSUHL)” has the ability to identify the lymph node accurately and outperform state-of-the-art methods on our collected real clinical dataset.

The main contributions of this paper are summarized as follows:

1. We propose a framework—CSUHL to tackle the task of identifying the lymph node, focusing on the uncertainty measurement of clinical CT imaging, as well as the cost sensitive hypergraph learning for identifying.
2. On our collected real clinical dataset, we conduct extensive experiments to demonstrate the proposed method consistently outperforms state-of-the-art methods across-the-board, relatively improving the performance on the accuracy (ACC), sensitivity (SEN), specificity (SPEC), Balance (BAC) by up to 3.90, 8.00, 2.02, and 4.95%, respectively, compared with the previous best method.

## 2. Materials and Methods

**Figure 2:**
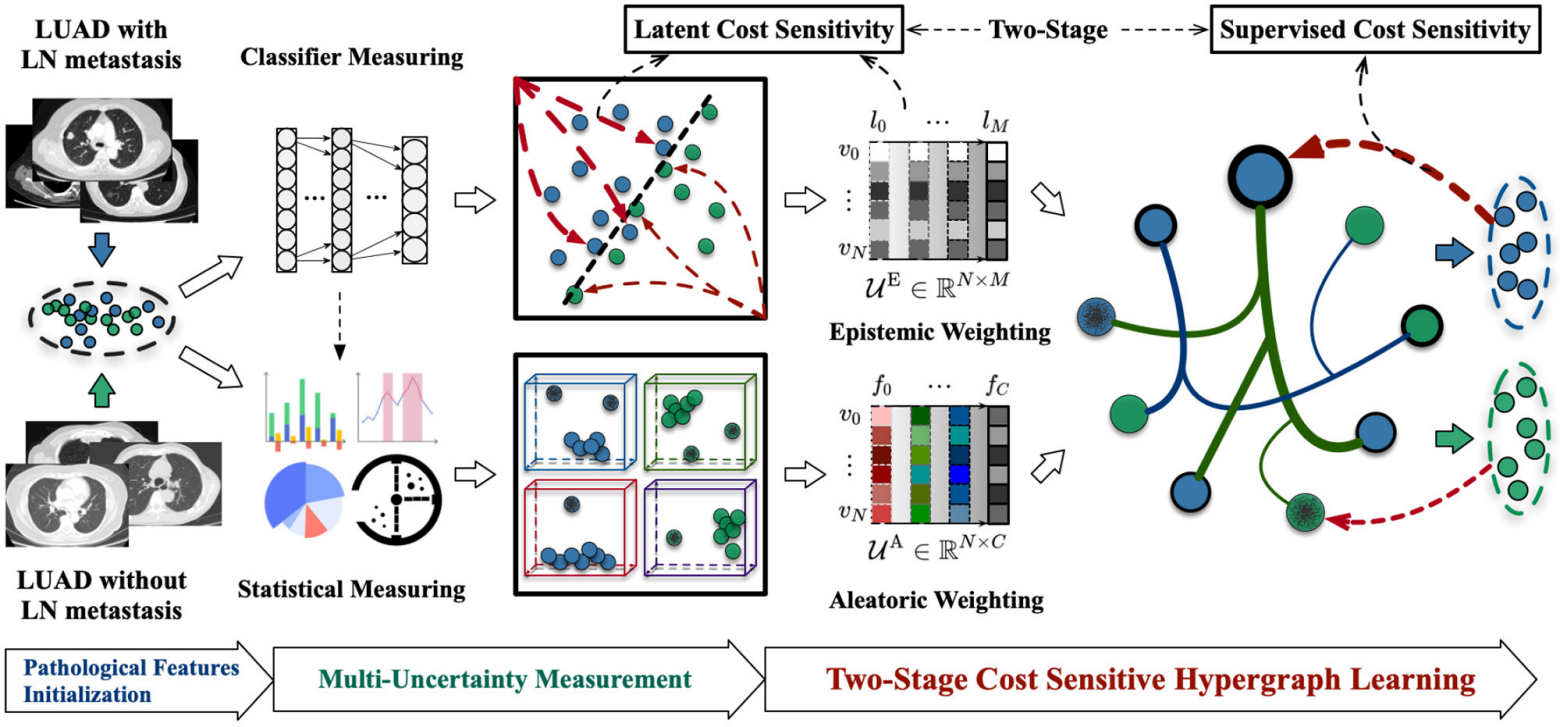
Illustration of our proposed Cost-Sensitive Uncertainty Hypergraph Learning (CSUHL) for identification of the LUAD with lymph node metastasis cases with CT imaging.

In this section, we will first introduce the materials we collected and the processing for extracting the initial features in details. Then we will illustrate our proposed framework—“Cost-Sensitive Uncertainty Hypergraph Learning (CSUHL),” as shown in Figure 2, which is composed of three steps, *i.e*., “Pathological Features Initialization,” “Multi-Uncertainty Measurement,” and “Two-Stage Cost Sensitive Hypergraph Learning,” respectively.

### 2.1. Materials and Preprocessing

In this study, a total of 61 CT images were collected, including 35 from lymph node negative patients and the rest 26 from lymph node positive patients. These images were provided by the China-Japan Friendship Hospital. All the cases were acquired from January 2017 to March 2019. The CT scanners used in this study include Aquilion ONE from TOSHIBA, MEDICAL System Revolution from GE, and SOMATOM Definition Flash from SEMENS. The CT protocol here includes: 120KV, reconstructed CT thickness is 1mm, and breath-hold at full inspiration. All images were de-identified before sending for analysis. This study was approved by the Institutional Review Board. Written informed consent was waived due to retrospective nature of the study.

### 2.2. Cost-Sensitive Uncertainty Hypergraph Learning

#### 2.2.1. Pathological Features Initialization

In this stage, we extract the initial features from the patient's CT images, consisting of regional features and radiomics features. We first apply the deep learning pre-trained method, named VB-Net (31), to segment the left/right lung, 5 lung lobes, 18 lung segments and infection lesions for each CT image in the portal software. In the expression of regional features, we generate a feature with a dimension of ℝ^96^ for each patient, expressing features such as the count of infected lesions and the mean value of lesion area. When extracting the radiomics features, we generated a feature with a dimension of ℝ^93^ for each patient, which means first-order intensity statistics and texture features. In the end, we concatenate the regional features and radiomics features obtained above to obtain an overall feature with a dimension of ℝ^*C*^ (*C* = 191) representing patient information.

#### 2.2.2. Multi-Uncertainty Measurement

As shown in Figure 2, there are two types of uncertainty measurement in our method, namely “Epistemic Weighting” and “Aleatoric Weighting,” respectively. The epistemic uncertainty refers to the inability of model for classifying the lymph node involvement in patients with LUAD. We utilize the general Multilayer Perceptron (MLP) Neural Network with the dropout variation inference to classify the data based on the initialized features. Illustrated as the “Epistemic Weighting” module in Figure 2, denoted as $U^{E}\in {\mathbb{R}}^{N\times M}$, the effect of the dropout can be attributed to imposing a Gaussian distribution on each layer during the inference stage. For *N* samples, there are *M* layers that, respectively, generate the epistemic uncertainty weights in different levels. For the *M* layers, each case with x^*^ features, is predicted for *K* times, the final epistemic weight for each case is calculated using the variance of these *K* values, formulated as Equation (1):


(1) $$\begin{array}{l} U_{q\left({\text{y}}^{*}|{\text{x}}^{*}\right)}^{E}\left({\text{y}}^{*}\right)=\frac{1}{KM}\overset{K}{\underset{k=1}{\sum }}\overset{M}{\underset{l=1}{\sum }}{\hat{\text{y}}}_{\left(l\right)}^{*}\left({\text{x}}^{*},\omega^{k}\right) \end{array}$$

where *i* denotes the *i*th sample and *k* denotes the *k*th test with dropout. (*l*) denote the *l*th layer of the constructed MLP model. ${\hat{\text{y}}}^{*}$ denotes the corresponding output of the input x^*^. $\omega^{k}={\left\{{\text{W}}_{i}\right\}}^{k}$ denotes the trainable variables for a model at the *k*th time.

We adopt a statistical measuring method to generate the aleatoric uncertainty weights $U^{A}$. As shown in Figure 2, the dimension of aleatoric uncertainty weights is ℝ^*N* × *C*^, where *N* and *C* denotes the number of samples and the scale of features, respectively. For each feature, we estimate the weights of aleatoric uncertainty by minimizing the Kullback-Leibler (KL) divergence (32–34) between the standard feature distribution and the predicted features. The detailed theoretical derivation and demonstration of calculation can be found in UVHL (23) and the main formulation is following:


(2) $$\begin{array}{l} U^{A}\left({\text{x}}_{i}\right)=\sigma_{\Theta }^{2}\left({\text{x}}_{i}\right)=exp\left(\alpha_{\Theta }\left({\text{x}}_{i}\right)\right) \end{array}$$

where $\sigma_{\Theta }^{2}$ denotes the predicted variance. To avoid the potential division by zero, α_Θ_(**x**) is the replacement of $\log\sigma_{\Theta }^{2}\left(\text{x}\right)$. Therefore, $\alpha_{\Theta }:{\mathbb{R}}^{191}\mapsto {\mathbb{R}}^{1}$ is the module to yield the aleatoric uncertainty score for each case.

#### 2.2.3. Two-Stage Cost Sensitive Hypergraph Learning

To identify the LUAD cases with higher sensitivity, we design a two-stage cost sensitive hypergraph learning in the final step of our framework. Given the vertices with initialized pathological features as well as the corresponding two types of uncertainty weights, we sequentially construct the uncertainty-vertex hypergraph and conduct cost-sensitive hypergraph learning.

When constructing the uncertainty-vertex hypergraph, we take each vertex v∈V denoting one sample with the corresponding two types of uncertainty weights $U^{E}$ and $U^{A}$. We use V to denote the vertex set, E denoting the hyperedges set, and **W** denoting the pre-defined matrix of hyperedge weights. We adopt the k-nearest neighbors algorithm (KNN) to define the relationships for each vertex. There are two groups of hyperedges, respectively, stand for the regional features and radiomics features, denoted as $E_{reg}$ and $E_{rad}$, which are represented by the corresponding incidence matrices ${\text{H}}_{reg}\in {\mathbb{R}}^{N\times |E_{reg}|}$ and ${\text{H}}_{rad}\in {\mathbb{R}}^{N\times |E_{rad}|}$. The final combined global incidence matrix $\text{H}\in {\mathbb{R}}^{N\times \left(\left|E_{reg}|+|E_{rad}\right|\right)}$ can be formulated as Equation (3).


(3) $$\begin{array}{l} \text{H}\left(v_{i},e_{j}\right)=\{\begin{array}{cc} U_{i}^{A}+U_{i}^{E}, & v_{i}\in e_{j},e_{j}\in \left[{\text{H}}_{reg}||{\text{H}}_{rad}\right]\\ 0, & v_{i}\notin e_{j},e_{j}\in \left[{\text{H}}_{reg}||{\text{H}}_{rad}\right] \end{array} \end{array}$$

where [·||·] denotes the concatenating operation between two matrices. Finish constructing the hyperedges, the uncertainty-vertex hypergraph can be denoted as G=〈V,E,H,W,U〉, where **U** is the summary matrix of $U^{A}$ and $U^{E}$.

There are two stages of operating the cost sensitivity, namely latent cost sensitivity and supervising cost sensitivity. When measuring the epistemic uncertainty weights in the stage of “Multi-Uncertainty Measurement,” we design the first latent cost sensitivity by the modified cross-entropy loss function, formulated as follows:


(4) $$\begin{array}{ll} L & =\frac{1}{N}\overset{N}{\underset{i}{\sum }}-\left[\lambda \cdot y_{i}\cdot \log\left(p_{i}\right)+\left(1-\lambda \right)\cdot \left(1-y_{i}\right)\cdot \log\left(1-p_{i}\right)\right] \end{array}$$

where *y*_*i*_ denotes the label of *i*th sample, whose value is 0 or 1 for negative case and positive case. λ ∈ (0, 1) is the parameter to represent the degree of cost sensitivity, whose value larger the more sensitivity. The other cost sensitivity for supervising is designed in the procedure of hypergraph learning, formulated as:


(5) $$\begin{array}{l} Q_{\text{U}}\left(\text{F}\right)=\arg\underset{\text{F}}{\min}\left\{\Omega \left(\text{F}\right)+\psi {\tilde{R}}_{emp}\left(\text{F}\right)\right\} \end{array}$$

where Ω(·) and ${\tilde{R}}_{emp}\left(\cdot \right)$ denote the smoothness regularizer function and the cost-sensitive empirical loss term, respectively. The hypergraph Laplacian matrix is $\Theta_{\text{U}}={\text{D}}_{v}^{-\frac{1}{2}}\text{H}\text{W}{\text{D}}_{e}^{-1}{\text{H}}^{\text{T}}{\text{D}}_{v}^{-\frac{1}{2}}$. The smoothness regularizer function is formulated as:


(6) $$\begin{array}{l} \Omega \left(\text{F},V,\text{U},E,\text{W}\right)=tr\left({\text{F}}^{⊤}\left({\text{U}}^{⊤}-{\text{U}}^{⊤}\Theta_{\text{U}}\text{U}\right)\text{F}\right) \end{array}$$

The cost-sensitive empirical loss term is designed as:


(7) $$\begin{array}{l} {\tilde{ℛ}}_{emp}(F,U)=\sum_{k=1}^{K}[\lambda \|F(:,k)-Y(:,k){\|}_{y=1}^{2}\\ \;+(1-\lambda )\|F(:,k)-Y(:,k){\|}_{y=0}^{2}] \end{array}$$

where **F**(:, *k*) is the *k*_*th*_ column of **F**. The λ in Equation (7) is same with the role in Equation (1).

The uncertainty vertex-weighted hypergraph loss function $R_{emp}\left(\cdot \right)$ can be further rewritten as:


(8) $$\begin{array}{l} \begin{array}{cc} {\tilde{R}}_{emp}\left(\text{F},\text{U},\lambda \right) & =\lambda ⊳\left[tr\left({\text{F}}^{⊤}{\text{U}}^{⊤}\text{U}\text{F}+{\text{Y}}^{⊤}{\text{U}}^{⊤}\text{U}\text{Y}-2{\text{F}}^{⊤}{\text{U}}^{⊤}\text{U}\text{Y}\right)\right] \end{array} \end{array}$$

Therefore, the target label matrix **F** can be obtained as:


(9) $$\begin{array}{l} \text{F}=\lambda ⊳\left[\psi {\left({\text{U}}^{⊤}-{\text{U}}^{⊤}\Theta_{\text{U}}\text{U}+\lambda {\text{U}}^{⊤}\text{U}\right)}^{-1}{\text{U}}^{⊤}\text{U}\text{Y}\right] \end{array}$$

where ⊳ denotes the degree of cost-sensitive λ operating on the following item, referring the effect in Equations 1 and 7. With the generated label matrix **F** ∈ ℝ^*n* × *K*^ (*K* = 2 in our task), the new coming testing case can be identified as LUAD or normal case accordingly.

## 3. Experiment

In this section, we will elaborate on the dataset, evaluation metrics, implementation, comparison methods, experimental results, and discussion.

### 3.1. Evaluation Metrics

In the experiment, we adopt six metrics to evaluate the accuracy of the model.

**(1)**. Accuracy (*ACC*) represents the proportion of correct predictions by the model and can be calculated as $ACC=\frac{TP+TN}{TP+TN+FP+FN}$. **(2)**. Sensitivity (*SEN*), **(3)**. Specificity (*SPEC*), **(4)**. Positive Predictive Value (*PPV*), and **(5)**. Negative Predictive Value (*NPV*), respectively, represent the proportion of correct predictions among the positive sample values, negative sample values, positive predicted values, and negative predicted values. The calculation formulas can be found in Table 1. **(6)**. Balance (*BAC*) represents the mean value of *SEN* and *SPEC*.

**Table 1:** The definition of the confusion matrix for identification of lymph node involvement.

	**Classify as lymph node involvement**	**Classify as non involvement**	
Lymph node involvement	True Positive (*TP*)	False Negative (*FN*)	$SEN=\frac{TP}{TP+FN}$
Non involvement	False Positive (*FP*)	True Negative (*TN*)	$SPEC=\frac{TN}{TN+FP}$
	$PPV=\frac{TP}{TP+FP}$	$NPV=\frac{TN}{TN+FN}$	

Sensitivity, known as true positive rate, represents the proportion of patients with lymph node involvement that are successfully detected in the task. Specificity represents the possibility of patients without lymph node involvement that are excluded. Ideally, the model with both high sensitivity and high specificity is what we most hope for, but in practice, there is a trade-off between these two indicators. Compared with specificity, higher sensitivity basically possesses greater practical value.

### 3.2. Implementation

The entire dataset contains 61 CT images, of which 26 are lymph node involvement and the remaining 35 are on the contrary, are randomly partitioned into 10 subsets when comparing our model with the comparison models. In the task, the cross-validation process is performed 10 times, each time a subset is selected as the validation set, and the rest as the training set. To reduce the impact of random data on the results, the value of each metric in the experiment is an average of 10 times, and the standard deviation is reported as a comparison. To prevent inductive bias, each dimension of the training set features is normalized to [0, 1] using its own mean and variance and samples in validation set utilize the same parameters to normalize.

The uncertainty score $U_{i}$ of each sample and the uncertainty measurement model are generated by the overall training set. The algorithm used to construct the incidence matrix of hypergraph is K-nearest neighbors (KNN), which leads the choice of parameter K to affect the effect. However, choosing K is not a easy job. A hyperedge from a large K connects too many modes, which may over-describe the relationship between the data and generate noise. On the contrary, a hyperedge with small K means that the number of connected nodes is small, which limits the exploration ability of the high-order relationship of hypergraph and not obtain full information. We conduct a strategy to learn the proper parameter K automatically here. We put 2 to 20 in the candidate pool of K to select the applicable K for the task. In one training and testing, we cross-validate the training data 10 times for each K in the candidate pool to obtain prediction values on different K. The K with the highest predicted score will be used in testing, so the whole process is the automatic selection of K. The total training time for each fold is about 1 h, while the testing time is extremely fast, only taking about 5 s.

### 3.3. Comparison Methods

The following methods are compared by our experiment:

1. Support Vector Machine (SVM) (35): It is a linear classifier that uses supervised learning to perform two-class classification of data. It relies on the convex quadratic programming problem to separate the samples correctly and away from the classification hyperplane.
2. Transductive Hypergraph Learning(tHL) (36): It is an algorithm for hypergraph embedding and transduction inference, mainly extending the spectral clustering technology of graphs to hypergraphs.
3. Multilayer Perceptron (MLP) Neural Network (37, 38): It contains a multi-layer feedforward neural network that maps multiple inputs to a single output.
4. GNN (39): It is a basic graph neural network that applies the local first-order approximation of the spectral graph convolution to determine the convolutional network structure for semi-supervised classification.
5. UVHL (23): It is an uncertainty vertex-weighted hypergraph learning method, which can reduce the problems caused by noisy data and confusing cases with clinical or imaging features.
6. B-GNN (40): It is a binary graph convolutional network, in which some floating-point operations are replaced by binary operations to achieve inference acceleration. A back-propagation method based on gradient approximation is used to train the binarized graph convolutional layer.
7. BC-GNN (40): It is an improved version of B-GNN, adding a cost-sensitive loss function.

### 3.4. Experimental Results

**Figure 3:**
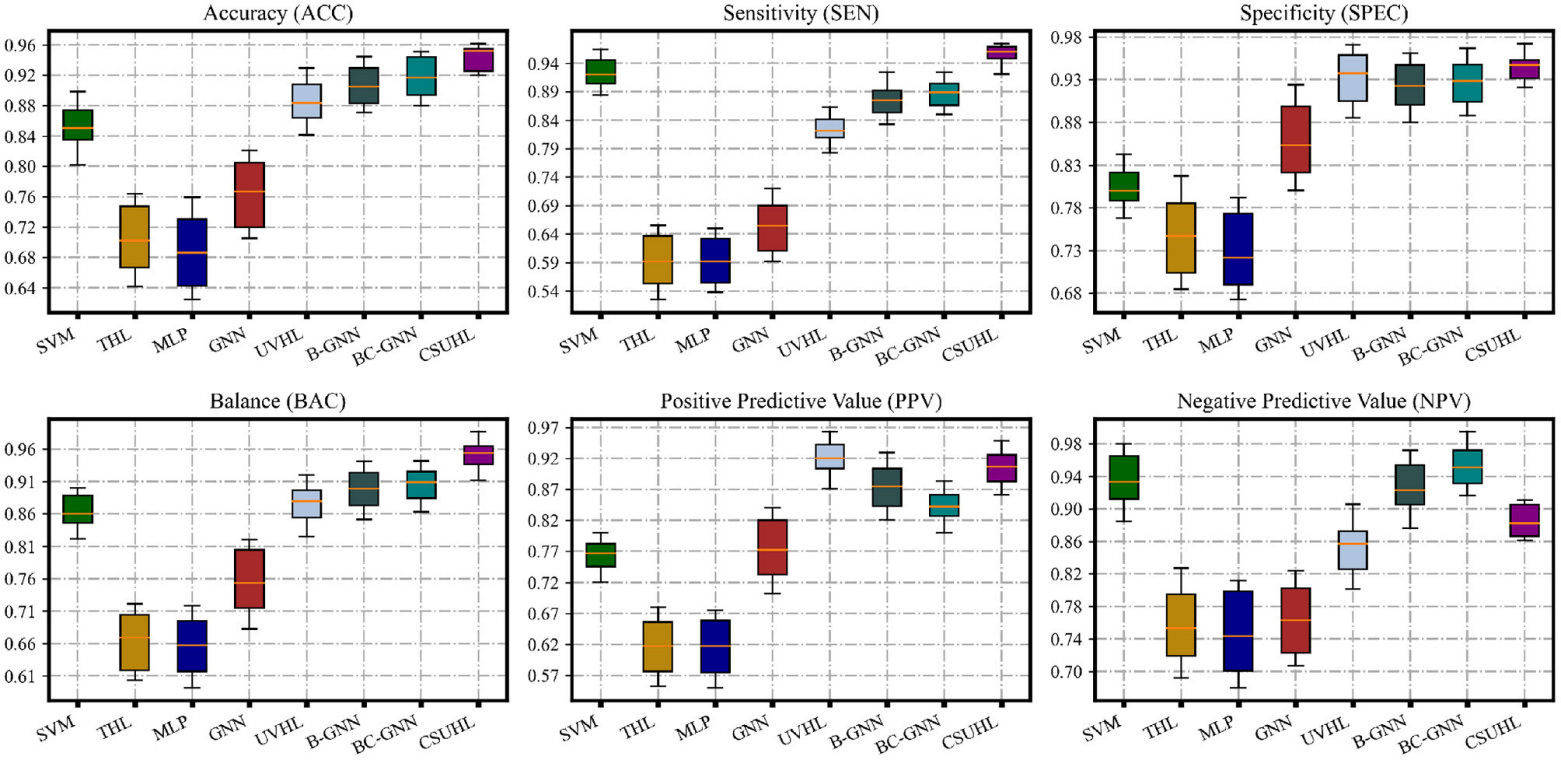
The statistic performance of CSUHL and other compared methods. The results show that CSUHL outperforms other methods for ACC, SEN, SPEC, and BAC consistently.

The experimental mean results and phenomena of our model and compared models can be seen in Figure 3; Table 2, and the following can be observed:

1. Comparing all methods, our proposed model is in a leading position in various metrics. Compared with non-graph-based methods (SVM and MLP), our models has a great lead. There are 12.04 and 38.89% improvements in ACC metric for the two, respectively, which shows that hypergraph has the ability to describe the correlation and handle this task.
2. In the GNN-based methods, compared with GNN, B-GNN, BC-GNN in ACC, the improvement is 24.22, 5.26, and 3.90%, respectively, which proves that our methods can describe complex associations better.
3. Compared with hypergraph-based methods such as THL and UVHL, the model has 35.59 and 7.82% rises on the acc, respectively, which benefits from the uncertainty measurement and multiple loss.
4. Except for ACC, our method is the only one that exceeds and close to 95% on SEN and SPEC, respectively, which has practical value for actual medical diagnosis.
5. From Figure 3, it can be observed from the standard deviation that our model has more stable results compared to other comparison methods, which shows that our model provides more reliable and robust prediction results.

**Table 2:** Prediction accuracy comparison of different methods on our collected LUAD dataset.

**Methods**		**ACC**		**SEN**		**SPEC**		**BAC**		**PPV**		**NPV**	
**SVM**	(*p-value*)	0.85000	_2.324*e*−5_	0.92000	_5.624*e*−4_	0.80000	_1.824*e*−4_	0.86000	_6.815*e*−5_	0.76667	_0.0498*e*−5_	0.93333	_6.781*e*−5_
**THL**	(*p-value*)	0.70238	_1.173*e*−5_	0.59167	_1.438*e*−6_	0.74667	_4.235*e*−4_	0.66917	_1.037*e*−4_	0.61667	_0.1.237*e*−5_	0.75333	_3.283*e*−6_
**MLP**	(*p-value*)	0.68571	_6.734*e*−4_	0.59167	_3.568*e*−5_	0.72167	_8.967*e*−3_	0.65667	_2.358*e*−4_	0.61667	_8.845*e*−4_	0.74333	_2.781*e*−5_
**GNN**	(*p-value*)	0.76667	_4.891*e*−4_	0.65385	_6.784*e*−4_	0.85294	_3.578*e*−4_	0.75339	_3.567*e*−4_	0.77273	_9.487*e*−4_	0.76316	_7.034*e*−4_
**UVHL**	(*p-value*)	0.88333	_2.346*e*−3_	0.82143	_7.624*e*−4_	0.93750	_6.78*e*−4_	0.87946	_1.895*e*−3_	**0.92000**	-	0.85714	_3.181*e*−4_
**B-GNN**	(*p-value*)	0.90480	_7.823*e*−3_	0.87500	_2.135*e*−3_	0.92300	_7.895*e*−3_	0.89900	_8.233*e*−3_	0.87500	_9.356*e*−3_	0.92300	_9.392*e*−3_
**BC-GNN**	(*p-value*)	0.91667	_4.721*e*−2_	0.88889	_1.468*e*−3_	0.92857	_2.568*e*−2_	0.90873	_9.134*e*−3_	0.84211	_7.804*e*−3_	**0.95122**	-
**CSUHL**	(*std*)	**0.95238** ^**†**^	*_±0.0346_*	**0.96000** ^**†**^	*_±0.0596_*	**0.94737** ^**†**^	*_±0.0277_*	**0.95368** ^**†**^	*_±0.0150_*	0.90654	*_±0.0286_*	0.88235	*_±0.0735_*

*For each 10-fold, we compute the accuracy of the proposed method on testing data, and compare them with those of CSUHL via paired t-test to generate the p-values for each metric. (“†” denotes significance level is reached as p−value < 0.05). The bold values represent the best values of the indicators in each set of experiments*.

## 4. Discussion

To evaluate the effectiveness of different uncertainty and different hypergraphs and different cost sensitivity, in this section, we conduct ablation experiments, respectively, to determine the contribution of each component.

### 4.1. Study on Multi-Uncertainty

To evaluate the effectiveness of different uncertainty, we conduct an ablation study, which uses aleatoric uncertainty or epistemic uncertainty, respectively.

#### 4.1.1. Aleatoric Uncertainty

Denoted as $U^{A}$, the results of using aleatoric uncertainty individually are shown in row 1 of Table 3.

**Table 3:** Prediction accuracy comparison of different methods on our collected LUAD dataset.

**Methods**		**ACC**		**SEN**		**SPEC**		**BAC**		**PPV**		**NPV**	
**1) Aleatoric Uncertainty** ($U^{A}$)	(*std*)	0.88525	_±0.1845_	0.92308	_±0.2451_	0.85714	_±0.1684_	0.89011	_±0.1795_	0.82759	_±0.0781_	**0.93750**	_±0.1864_
**2) Epistemic Uncertainty** ($U^{E}$)	(*std*)	0.85246	_±0.0351_	0.88462	_±0.0763_	0.82857	_±0.0374_	0.85659	_±0.0746_	0.79310	_±0.0890_	0.90625	_±0.0785_
**3) CSUHL** ($U^{A}+U^{E}$)	(*std*)	**0.95238** ^**†**^	*_±0.0346_*	**0.96000** ^**†**^	*_±0.0596_*	**0.94737** ^**†**^	*_±0.0277_*	**0.95368** ^**†**^	*_±0.0150_*	**0.90654**	*_±0.0286_*	0.88235	*_±0.0735_*
**4) Regional Hypergraph** ($G_{reg}$)	(*std*)	0.80328	_±0.0567_	0.80769	_±0.978_	0.80000	_±0.1643_	0.80385	_±0.0776_	0.75000	_±0.1347_	0.84848	_±0.1613_
**5) Radiomics Hypergraph** ($G_{rad}$)	(*std*)	0.90164	_±0.0891_	0.92308	_±0.0346_	0.88571	_±0.0917_	0.90440	_±0.0176_	0.85714	_±0.0783_	**0.93939**	_±0.0635_
**6) CSUHL** ($G_{reg}+G_{rad}$)	(*std*)	**0.95238** ^**†**^	*_±0.0346_*	**0.96000** ^**†**^	*_±0.0596_*	**0.94737** ^**†**^	*_±0.0277_*	**0.95368** ^**†**^	*_±0.0150_*	**0.90654**	*_±0.0286_*	0.88235	*_±0.0735_*
**7) Latent Cost Sensitivity**	(*std*)	0.86885	_±0.0678_	0.88462	_±0.0341_	0.85714	_±0.0867_	0.87088	_±0.1456_	0.82143	_±0.1034_	0.90909	_±0.0918_
**8) Supervising Cost Sensitivity**	(*std*)	0.91803	_±0.0451_	0.92308	_±0.0813_	0.91429	_±0.0561_	0.91868	_±0.0971_	0.88889	_±0.0936_	**0.94118**	_±0.0771_
**9) CSUHL**	(*std*)	**0.95238** ^**†**^	*_±0.0346_*	**0.96000** ^**†**^	*_±0.0596_*	**0.94737** ^**†**^	*_±0.0277_*	**0.95368** ^**†**^	*_±0.0150_*	**0.90654**	*_±0.0286_*	0.88235	*_±0.0735_*

*For each 10-fold, we compute and report the average performance of the proposed method on testing data. The bold values represent the best values of the indicators in each set of experiments*.

We can find out that the use of both types of uncertainty brings about 7.58, 4, and 10.53% growth in ACC, SEN, and SPEC, respectively, than using aleatoric uncertainty. It is worth mentioning that even with only aleatoric uncertainty, the model is still higher than most comparison methods in ACC and better than other methods in SEN according to Table 2.

More specifically, the pathological features with higher aleatoric uncertainty weights are consistent with the clinical experience, such as the distribution of different nodules, *i.e*., lobulated nodules, spiculate nodules, and globular nodules.

#### 4.1.2. Epistemic Uncertainty

Denoted as $U^{E}$, the results of using epistemic uncertainty individually are shown in row 2 of Table 3.

It can be observed using epistemic uncertainty alone lags behind 10.49, 7.85, and 12.54 than CSUHL in acc, sen, and spec metrics, respectively. Compared with the results of using aleatoric uncertainty, the indicators using epistemic uncertainty are lower, indicating that aleatoric uncertainty plays a greater role in our model. The combination of the two uncertainties is better than the single-use, which proves the effectiveness of multi-uncertainty.

### 4.2. Study on Types of Hypergraph

To evaluate the effectiveness of different hypergraphs, we conduct an ablation study, using regional hypergraph or radiomics hypergraph, which use regional features and radiomics features from CT, respectively.

#### 4.2.1. Regional Hypergraph

Denoted as $G_{reg}$, the results of using regional hypergraph individually are shown in row 4 of Table 3.

The regional hypergraph only has an accuracy rate of about 80%, and the same for SEN and SPEC, indicating that only extracting the regional features of CT has little effect and the regional hypergraph cannot provide accurate correlation information.

#### 4.2.2. Radiomics Hypergraph

Denoted as $G_{rad}$, the results of using radiomics hypergraph individually are shown in row 5 of Table 3.

The results of radiomics hypergraph are much better than the former, with ACC and SEN exceeding 90%, although there is still a gap in the combination of two hypergraphs. It can be found that the results in radiomics hypergraph have better sensitivity than specificity, which proves that the radiomic hypergraph has more advantages in identifying lymph node involvement. The combined hypergraph is higher in all indicators than when used alone, showing that it has the ability to utilize a variety of different features.

### 4.3. Study on Cost Sensitivity

To evaluate the effectiveness of different cost sensitivity, we conduct an ablation study, which uses latent cost sensitivity or supervising cost sensitivity, respectively.

#### 4.3.1. Latent Cost Sensitivity

The results of using latent cost sensitivity individually are shown in row 7 of Table 3.

When only latent cost sensitivity is used, it is equivalent to not using supervising cost sensitivity. As a result, the hypergraph information cannot be captured in order that various indicators are significantly reduced.

#### 4.3.2. Supervising Cost Sensitivity

The results of using supervising cost sensitivity individually are shown in row 8 of Table 3.

Compared with the combination of the two cost sensitivity, the supervising cost sensitivity has an accuracy disparity of about 3.61%, but it is higher than latent cost sensitivity. It should be noticed that cost sensitivity of using only supervising is the highest among the three on NPV, indicating that the true label is mostly negative in the samples identified as non lymph node involvement. In general, using two cost sensitivities together is better than using one of them, proving the effectiveness of cost sensitivity component.

## 5. Conclusion

In this paper, we propose a cost-Sensitive Uncertainty Hypergraph Learning (CSUHL) to identify lung adenocarcinoma (LUAD) cases with lymph node (LN) metastasis from the cases without lymph node (LN) metastasis. Confronting the challenging issues from the shortage of high-quality data and unreliable sensitivity of diagnosis, our proposed method employs three stages, namely “Pathological Features Initialization,” “Multi-Uncertainty Measurement,” and “Two-Stage Cost Sensitive Hypergraph Learning” to represent the complex clinical information and formulate the high-order data correlation among the known LUAD with LN metastasis cases and the LUAD without LN metastasis cases. Through the epistemic and aleatoric uncertainty as well as the two types of cost sensitivity (latent and supervising), our method is capable of outperforming state-of-the-art methods on our collected LUAD dataset across the board.

In future work, we will further investigate the practical limitations on the computer-aid-diagnosis (CAD), such as enhancing the speed of inference, transferring the model to learn, and predicting the other related downstream clinical tasks.

## Data Availability Statement

The raw data supporting the conclusions of this article will be made available by the authors, without undue reservation.

## Ethics Statement

The studies involving human participants were reviewed and approved by Clinical Research Ethics Committee of China-Japan Friendship Hospital. The patients/participants provided their written informed consent to participate in this study.

## Author Contributions

QM and DD: study design. QM, QY, YZ, and CL: data collection. JY, JZ, and DD: data analysis. JZ and DD: supervision. JY and DD: manuscript writing. All authors contributed to the article and approved the submitted version.

## Funding

This study was supported by the Peking science and technology fund (No. Z191100006619008) and Elite Medical Professionals project of China-Japan Friendship Hospital (No. ZRJY2021-GG02).

## Conflict of Interest

JZ is employed by Tencent AI Lab, China. The remaining authors declare that the research was conducted in the absence of any commercial or financial relationships that could be construed as a potential conflict of interest.

## Publisher's Note

All claims expressed in this article are solely those of the authors and do not necessarily represent those of their affiliated organizations, or those of the publisher, the editors and the reviewers. Any product that may be evaluated in this article, or claim that may be made by its manufacturer, is not guaranteed or endorsed by the publisher.
